# Low Insulin-Like Growth Factor-1 Level in Obesity Nephropathy: A New Risk Factor?

**DOI:** 10.1371/journal.pone.0154451

**Published:** 2016-05-03

**Authors:** Ioana Bancu, Maruja Navarro Díaz, Assumpta Serra, Marisa Granada, Dolores Lopez, Ramon Romero, Josep Bonet

**Affiliations:** 1 Department of Nephrology, Hospital Universitari Germans Trias i Pujol, Badalona, Barcelona, Spain; 2 Universitat Autònoma de Barcelona, Barcelona, Spain; 3 REDinREN, Madrid, Spain; 4 Department of Biochemistry, Hospital Universitari Germans Trias i Pujol, Badalona, Barcelona, Spain; 5 Department of Pathology, Hospital Universitari Germans Trias i Pujol, Badalona, Barcelona, Spain; Hospital Universitario de La Princesa, SPAIN

## Abstract

**Introduction:**

IGF-1 (insulin-like growth factor-1) is a hormone involved in cell growth and other important processes. In the kidney, IGF-1 has a stimulating effect, increasing the blood flow and glomerular filtration rate. Although many experimental animal studies regarding the role of IGF-1 in the kidney have been conducted, few human studies are available in the literature. Obesity is a cause of renal failure, and several glomerular lesions associated with obesity have been described. However, no studies regarding the levels of IGF-1 in morbidly obese patients with renal injury associated with obesity have been conducted.

**Aim:**

To determine the serum IGF-1 concentrations in morbidly obese patients with normal renal function but with different types of early obesity-related glomerular lesions and to evaluate the possible relationship between IGF-1 and the presence of renal lesions.

**Methods:**

Eighty morbidly obese patients with renal biopsy, including 11 patients with no evidence of renal lesion, 17 patients with single glomerulomegaly, 21 patients with single podocyte hypertrophy, 10 patients with glomerulomegaly and podocyte hypertrophy, 5 patients with focal segmental hyalinosis, and 16 patients with increased mesangial matrix and/or mesangial proliferation, participated in this study. Biological parameters, including serum IGF-1 concentrations with the standard deviation score for age (SDS-IGF-1), were determined for all patients.

**Results:**

Eighty patients (50 women and 30 men) with a mean BMI of 52.63 ± 8.71 and a mean age of 42.40 ± 9.45 years were included in this study. IGF-1, IGF-1 SDS and IGF-1BP3 levels according to the renal injury were compared (normal glomeruli: IGF-1 = 190.17 ± 72.46; glomerulomegaly: IGF-1 = 122.3 ± 50.05; podocyte hypertrophy: IGF-1 = 119.81 ± 60.34; focal segmental hyalinosis: IGF-1 170.98 ± 100.83, increased mesangial matrix and/or mesangial proliferation: IGF-1 117.73 ± 63.87). Statistically significant differences were observed between serum levels of IGF-1 and between the levels of SDS-IGF-1 by comparing the group without glomerular lesion with the group formed by patients with any type of glomerular injury. Logistic regression analysis was performed, with the dependent variable defined as the glomerular injury. In the multivariate analysis, only SDS-IGF-1 was associated with glomerular injury, and low levels of IGF-1 SDS were a risk factor for kidney injury.

**Conclusions:**

Our study demonstrates that low IGF-1 serum levels are associated with renal lesions in morbidly obese patients without overt clinical renal manifestations.

## Introduction

Insulin-like growth factor 1 (IGF-1) is a peptide hormone synthesized mainly by liver mesenchymal cells under the control of growth hormone (GH) and secreted into the blood. The plasma IGF-1 concentrations depends primarily on the GH levels, nutritional status, and physical activity level [1,2]. IGF-1 promotes growth and differentiation in a variety of tissues and also maintains structural integrity, inhibits apoptosis, and exerts anti-inflammatory effects.

Insulin like growth factor 1 deficiency has been linked to increased risks of stroke and ischemic heart disease [3,4]. Further, low serum IGF-1 concentrations have been consistently observed in severely obese patients [5,6].

Insulin like growth factor 1 stimulates the kidney by increasing blood flow and the glomerular filtration rate (GFR) [7]. Many experimental animal studies have been performed to determine the effects of IGF-1 on renal function. However, the few human studies that have been conducted have yielded conflicting results. The serum IGF-1 is lower in children with chronic kidney disease (CKD) than in those with a normal GFR [8]. However, increases in the IGF-1 and IGF-1 binding protein 3 (IGFBP-3) levels have been observed in adult patients with a decreased estimated GFR [9], and low IGF-1 have been reported in adult patients with CKD and cachexia [10]. Obesity is a cause of CKD. We have previously reported the association of several glomerular lesions (increased mesangial matrix, mesangial cell proliferation, podocyte hypertrophy, glomerulomegaly, and focal and segmental glomerulosclerosis) with morbid obesity in patients without overt clinical renal manifestations [11]. However, no studies have investigated the IGF-1 level in morbidly obese patients and its association with renal lesions.

Our principal aim was to compare the serum IGF-1 concentrations in morbidly obese patients with normal renal function with or without early obesity-related glomerular lesions. We evaluated the association of the IGF-1 levels with the presence of different types of renal lesions and with several biochemical parameters.

## Material and Methods

We analyzed patients’ serum IGF-1 concentrations determined in a previous study performed by our research group [11]. One hundred thirty morbidly obese patients with an unknown history of kidney disease who had undergone open bariatric surgery (and renal biopsy at surgery) at our hospital between December 2001 and November 2005 were invited to participate in the study.

Of the 130 patients, 5 refused to participate, and 30 could not undergo renal biopsy or had insufficient renal tissue available for histological analysis. The remaining 95 patients were included in the previous study [11].

Of the 95 patients, 80 patients whose IGF-1 level was determined before bariatric surgery were included in the current study.

None of the selected patients had a history of renal disease or was currently on insulin therapy, oral antidiabetic treatment or lipid-lowering drugs. This study was approved by the ethics committee of our hospital, and all patients signed informed consent forms.

Blood samples were drawn from fasting patients at baseline, before bariatric surgery. Serum samples were kept frozen at -80°C until measurement of adiponectin (ADPN) concentrations. Urine samples were collected from all patients during the previous 24 h period. Plasma glucose, total cholesterol, triglyceride, creatinine and urinary creatinine levels were measured using a Cobas^®^ 711 Roche Diagnostics analyzer. Creatinine clearance was calculated using the following equation: 24 h urine (ml) x urinary creatinine concentration x 1000 / (plasma creatinine concentration x 1440 min). Proteinuria (24 h) was determined by a spectrophotometric method (pyrogallol red) and albuminuria (24 h) was measured by nephelometry. Serum ADPN concentrations were measured using a commercial radioimmunoassay (Linco Research Inc., St Louis, MO). The intra-assay coefficient of variation (CV) was below 6.2%, and the inter-assay CV was below 9.2%. High sensitivity C-reactive protein (HS-CRP) concentrations were determined using an ultrasensitive CRP test (N HS-CRP; Dade Behrin Marburg GmbH, Marburg, Germany), and the inter-assay CV was < 3.9%. The detection limit of the assay was 0.175 mg/L, and it was performed at a sample dilution of 1:20.

Serum total IGF-1 and IGFBP-3 levels were measured using a chemiluminescence immunoassay (Immulite 2000, Siemens Healthcare Diagnostics Products, UK). IGF-I was standardized against the World Health Organization/National Institute for Biological Standards and Control (WHO NIBSC) 1^st^ IRR 87/518 (1 ng/ml x 0.13 = 1 nmol/L; the sensitivity was 20 ng/mL) and the intra-assay and inter-assay CVs were < 3.9 and < 7.7%, respectively. IGFBP-3 was standardized against the WHO NIBSC Reagent 93/560 (1 μg/ml x 34.78 = 1 nmol/L). The sensitivity was 0.1 μg/mL, and the intra-assay and inter-assay CVs were < 4.6 and < 7.3%, respectively. The IGF-I/IGFBP-3 ratio was calculated on a molar basis (IGF-I nmol/L/IGFBP-3 nmol/L).

Standard deviation scores (SDS) were calculated according to an age-related reference population mean based on the data provided by the assay manufacturer for each IGF-1 level. Low IGF-1 was defined as an IGF-1 SDS of below -2.

Body mass index (BMI) was calculated as weight in kilograms divided by height in meters squared. Waist circumference was measured at the approximate midpoint between the lower margin of the last palpable rib and the top of the iliac crest. Blood pressure was determined with a standard mercury sphygmomanometer cuff of suitable size. Hypertension was defined as systolic blood pressure (SBP)≥ 140 mmHg and/or diastolic blood pressure (DBP) ≥ 90 mmHg. The diagnostic criteria for diabetes was a fasting plasma glucose levels of ≥ 7.0 mmol/L in two determinations and impaired fasting glucose, asdefined by a fasting serum glucose level of ≥ 5.6 and <7 mmol/L. Hypercholesterolemia was defined as a total serum cholesterol level of > 5.7; hypertriglyceridemia was defined as a triglyceride level of > 1.71 mmol/L. The GFR was estimated according to creatinine clearance measured in a 24 h urine sample (without correction for body surface area). Microalbuminuria was defined as the presence of 30 to 300 mg albumin in a 24 h urine sample and proteinuria was defined as the presence of > 300 mg protein or albumin.

The insulin resistance index was calculated using the homeostasis model assessment of insulin resistance (HOMA-IR) as (fasting insulin mU/L) × (fasting glucose mmol/L) / 22.

### Histological data

Renal biopsies were processed for histology as described in our previous study [11]. Glomerulomegaly was defined as a glomerular area of above the mean glomerular area of a control group plus two standard deviations. Podocyte hypertrophy was defined as podocyte enlargement with large nuclei and prominent nucleoli. Increased mesangial matrix was defined as mesangial sclerosis resulting in widening of the mesangium. Mesangial cell proliferation was defined as the presence of more than three mesangial cells surrounded by mesangial matrix in an intact glomerular segment. Focal segmental hyalinosis was defined as focal and segmental consolidation of the glomerular tuft by increased extracellular matrix, with obliteration of the capillary lumina.

Each biopsy was assessed as described above, and the presence of one or several histological lesions was registered.

### Statistical analyses

The data were first tested for a normal distribution using the Kolmogorov-Smirnov test. Normally distributed variables were expressed as the mean ± SD. Nonparametric variables were expressed as the median (25^th^ and 75^th^ percentiles). Associations of the IGF-1 level with the clinical, anthropometric, biochemical and pathological data were identified using Pearson’s correlation test, Student’s t-test and analysis of variance (ANOVA) as appropriate. Logistic regression models were used to assess the associations of the IGF-1 level with the biochemical, clinical and glomerular lesions.

All statistical analyses were conducted with SPSS statistical software package (version 15.0; SPSS, Chicago, IL). Statistical significance was considered at a p<0.05.

## Results

### Clinical, anthropometric and biochemical data

The data are summarized in Table 1.

**Table 1:** Anthropometric and biochemical characteristics.

Age (years)	42.40 ± 9.45
Gender (F)	62.5%
BMI (kg/m^2^)	52.63 ± 8.71
Creatinine (mmol/L)	81.11 ± 12.36
Creatinine Clearence (mL/min)	135.96 ± 40.95
HOMA-IR (%)	5.19 ± 3.38
HS-CRP (mg/L)	9.84 (5.32–17.20)
ADPN (mcg/mL)	5.72 ± 3.05
Microalbuminuria (mg/24 h)	25.65 (8.30–92.55)
IGF-1 (ng/mL))	134.15 ± 64.97
IGF-1 SDS	0.0006 (-1.52–0.77)

Fifty-three percent of the patients had arterial hypertension, 44% had hypercholesterolemia, 27% had hypertriglyceridemia. Twenty-one percent of patients exhibited impaired fasting glucose, and 18.5% presented with a fasting plasma glucose level of higher than 7 mmol/L but lower than 11 mmol/L in all cases. Only 4% exhibited a urinary albumin excretion rate of higher than 300 mg/24 h but lower than 500 mg/24 h in all cases.

### Histological data

Pathological examinations revealed no evidence of renal injury in 11 patients, and the presence of renal lesion in 69 patients. Of the 69 patients, glomerulomegaly alone was found in 17 patients, podocyte hypertrophy alone in 21, glomerulomegaly and podocyte hypertrophy in 10, focal segmental hyalinosis in 5, and increased mesangial matrix and/or mesangial proliferation in 16.

### Differences in clinical and biochemical data in extremely obese patients with and without renal lesion

We analyzed the differences in age, BMI, the glucose level, blood pressure, creatinine clearance, microalbuminuria, and insulin resistance between the groups with and without renal lesions (Table 2) and found significant differences in age and BMI between the two groups.

**Table 2:** Baseline characteristics of the groups with and without renal lesions.

Variable	Without renal lesion (n = 11)	With renal lesion (n = 69)	p value
Age	36.55±10.43	43.33±9.02	0.026
BMI	47.71±4.62	53.38±8.98	0.05
SBP	134.73±10.99	140.67±18.03	0.29
DBP	82.18±9.81	81.81±13.38	0.9
Glucose	5.55±1.17	5.96±1.71	0.45
HOMA-IR	5.1±3.38	5.2±3.42	0.94
Creatinine clearance	158.200±57.58	132.41±36.99	0.17
Microalbuminuria	52.41±7.91	73.35±11.9	0.54

### Associations between IGF-1 and clinical, anthropometric and biochemical data in extremely obese patients

According to the correlation analysis, IGF-1 levels are associated with age (r: -0,481, p < 0.0001), insulin level (r: 0.272, p = 0.014), and creatinine clearance (r: 0.361, p = 0.001).

No significant associations were observed between the IGF-1 level and BMI, the glucose level, the HOMA-IR value or the inflammatory parameters (HS-CRP and ADPN levels).

### IGF-1 levels and IGF-1 SDS according to the type of obesity related glomerular lesion

We consecutively compared the IGF-1 levels and IGF-1 SDS between the morbidly obese patients without a renal lesion and those with any type obesity related glomerular lesion.

**Fig 1:**
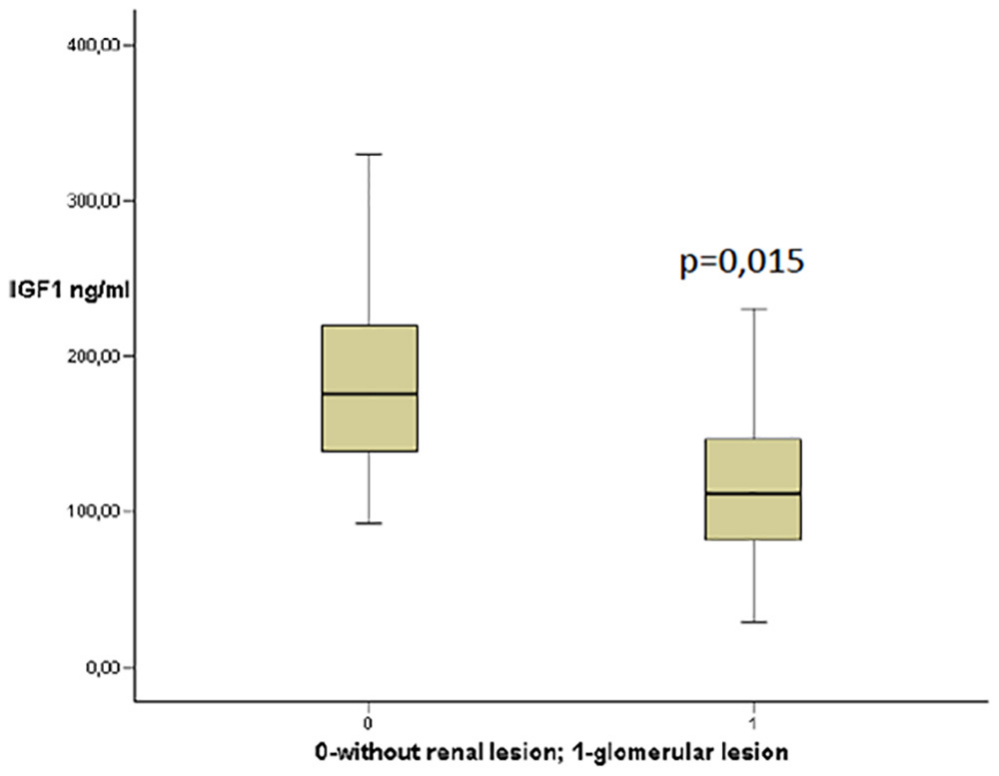
IGF-1 levels in patients with and without renal lesion. IGF-1 levels and IGF-1 SDS in patients without renal lesions were significantly different from patients (with n > 10) with different types of obesity related glomerular lesion.

The serum IGF-1 levels and IGF-1 SDS were significantly decreased in patients with any type of obesity related glomerular lesion (n = 69) compared with those without glomerular lesions (n = 11) (Fig 1). No significant differences were found in the IGFBP-3 level between the groups (with or without glomerular lesion).

**Fig 2:**
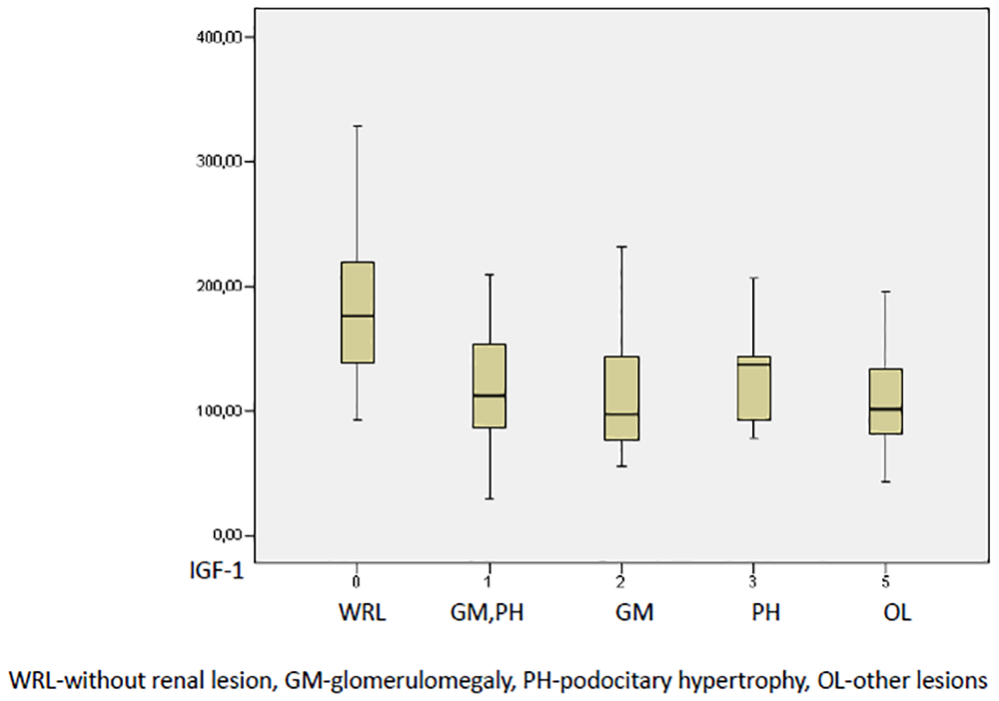
IGF-1 levels in patients without renal lesion and with different types of renal. Univariate and multivariate logistic regression models were used to analyze variables such as SBP, DBP, BMI, creatinine clearance, glucose, HOMA-IR, HS-CRP, and ADPN in our study population. The presence of a glomerular lesion was defined as the dependent variable. Univariate analysis revealed that the presence of a glomerular lesion was associated with a low IGF-1 SDS and BMI.

Patients with a renal lesion of any type had a lower IGF-1 level than those without a renal lesion (Table 3, Fig 2).

**Table 3:** IGF-1 levels and SDS in patients without renal lesions and in those with different types of lesions.

*variable*	*WRL (n = 11)*	*GM (n = 17)*	*GM*, *PH (n = 10)*	*PH (n = 21)*	*OL (n = 16)*	*p value*
*IGF-1*	*190*.*17 ± 72*.*46*	*122*.*3 ±0*.*05*	*122*.*94 ± 52*.*08*	*119*.*81 ± 60*.*34*	*117*.*73 ± 63*.*87*	*0*,*016*
*SDS-IGF-1*	*0*.*49 (0*.*25–1*.*46)*	*-0*.*05 (-1*.*54–0*.*85)*	*-0*.*21 (-1*.*99–0*.*59)*	*-1*.*32 (-2*.*28–0*.*416)*	*-0*,*822 (-1*,*88–0*,*24)*	*0*,*071*

WRL without renal lesion, GM glomerulomegaly, PH podocitary hypertrophy, OL other lesions

However, according to multivariate analysis, only a low IGF-1 SDS was associated with glomerular injury (Table 4).

**Table 4:** Logistic regression model for the presence of a glomerular lesion.

	Univariate		Multivariate	
	OR 95% CI	p value	OR 95% CI	p value
SBP	1.021(0.983–1.060)	0.29	-	-
DPB	0.99.8 (0.950–1.048)	0.998	-	-
BMI	1.132 (1.005–1.267)	0.043	-	-
Glucose	1.218(0.731–2.032)	0.449	-	-
Cr clearance	0.986 (0.972–1.001)	0.060	-	-
Insulin	0.895(0.927–1.047)	0.637	-	-
HS-CRP	1.037 (0.957–1.123)	0.367	-	-
ADPN	1.176 (0.864–1.600)	0.302	-	-
IGF-1 SDS	0.617 (0.396–0.960)	0.032	0.635(0.405–0994)	0.047

CI, confidence interval; OR, odds ratio; SBP, systolic blood pressure; DPB, diastolic blood pressure; BMI, body mass index; Cr, creatinine; HS-CRP, high-sensitivity C-reactive protein; ADPN, adiponectin

## Discussion

This is the first clinical study to demonstrate that a low IGF-1 level is associated with renal lesions in morbidly obese patients without clinical renal manifestations.

In our study, the serum IGF-1 level was found to be associated with age, the insulin level, and the creatinine clearance. The age-dependency of IGF-1 is well known; a negative correlation between the circulating IGF-1 level and age has been reported in previous studies [12,13]. Similar to our findings, a study of obese and insulin-resistant subjects has revealed a significant correlation between the IGF-1 level and GFR [14], however no data have been reported on glomerular lesions in this study.

The etiology of decreased IGF-1 in obese patients is thought to be multifactorial.

An increase in the IGF-1 level after weight loss has been demonstrated, suggesting that obesity-related IGF-1 deficiency is an acquired condition [15]. A low serum IGF-1 level may indicate its reduced production, and, notably, obese patients exhibit decreased GH secretion [16].

Insulin resistance is a well-known feature of obesity, and a low plasma IGF-1 concentration has been reported to be significantly associated with insulin sensitivity [17]. Obesity represents a state of chronic inflammation, and inflammation has been suggested to be related to renal lesions in extremely obese patients [18].

In addition, the IGF and inflammatory systems are closely linked; an inverse association between CRP and IGF has been previously demonstrated [19].

In our study morbidly obese patients with renal lesions had a lower IGF-1 level than those without renal lesions. Furthermore IGF-1 level was lower in the groups of patients with different types of obesity related glomerular lesion when comparing with those without renal lesion.

The physiological link between an obesity-related low IGF-1 level and the presence of renal lesions is not completely understood; however, it is widely accepted that kidney disease influences the IGF/GH axis [20,21]. Renal IGF-1 has been shown to originate from circulating and locally synthesized IGF-1, although locally produced renal IGF-1 has been shown to have no significant effect on kidney growth [22]. IGF-1 inhibits podocyte and mesangial cell apoptosis [23], and it is a potent mitogen for glomerular mesangial cells [24,25]. Growth hormone and IGF-1 deficiencies have been associated with decreases in glomerular filtration and renal plasma flow [26], although IGF-1 also seems to have GH-independent renal activity [27]. Animal studies have demonstrated generalized organ hypoplasia in IGF-1-deficient mice [28]. Further, decreased IGF-1 levels have been reported in diabetic [29], end-stage renal disease and hemodialysis patients [30,31].

In the recent literature, a role of IGF-1 as a therapeutic agent has also been discussed [32]. In animal studies, IGF-1 overexpression has been shown to improve the therapeutic action of mesenchymal stem cells against acute kidney injury [33]. In addition, prolonged treatment with recombinant human (rh) IGF-1 has been demonstrated to increase kidney size in hypophysectomized rats [34], while treatment of humans with rh IGF-1 increases glomerular filtration and kidney size and has specific anabolic effects on renal function, potentially delaying the onset of end-stage renal disease [35–38]

Interestingly, our patients did not present renal failure. Thus, a low IGF-1 level may be an early marker of the presence of occult renal lesions.

In our study, a lowIGF-1 SDS was associated with glomerular lesions in multivariate analysis. Our results suggest that a low IGF-1 SDS may be a risk factor for the development of renal lesion in extremely obese patients

A limitation of our study is the relatively small sample size. However, the characteristics of the included patients (morbidly obese with normal renal function and early diagnosis by renal biopsy performed during bariatric surgery) were unique; thus, it would have been very difficult to increase the sample size because renal biopsy is not clinically indicated in morbidly obese patients with normal renal function, and open bariatric surgery is no longer performed on these patients at our hospital.

Another limitation of our study was that our study group was limited to morbidly obese patients; therefore, our results are not applicable to patients with other degrees of obesity.

Further studies are required to clarify the pathogenic mechanisms underlying the involvement of IGF-1 in obesity-related nephropathy.

In conclusion, our findings demonstrate that a low IGF-1 serum level is associated to obesity related glomerular lesion in morbidly obese patients.

## Supporting Information

S1 Fig IGF-1 levels in patients with and without renal lesion. (PDF)

S2 Fig IGF-1 levels in patients without renal lesion and with different types of renal. (PDF)
