# Supplementary figures and images for: Low Insulin-Like Growth Factor-1 Level in Obesity Nephropathy: A New Risk Factor?
Source: PLoS One. 2016 May 3;11(5):e0154451. doi: 10.1371/journal.pone.0154451 (PMC4854474; doi:10.1371/journal.pone.0154451)

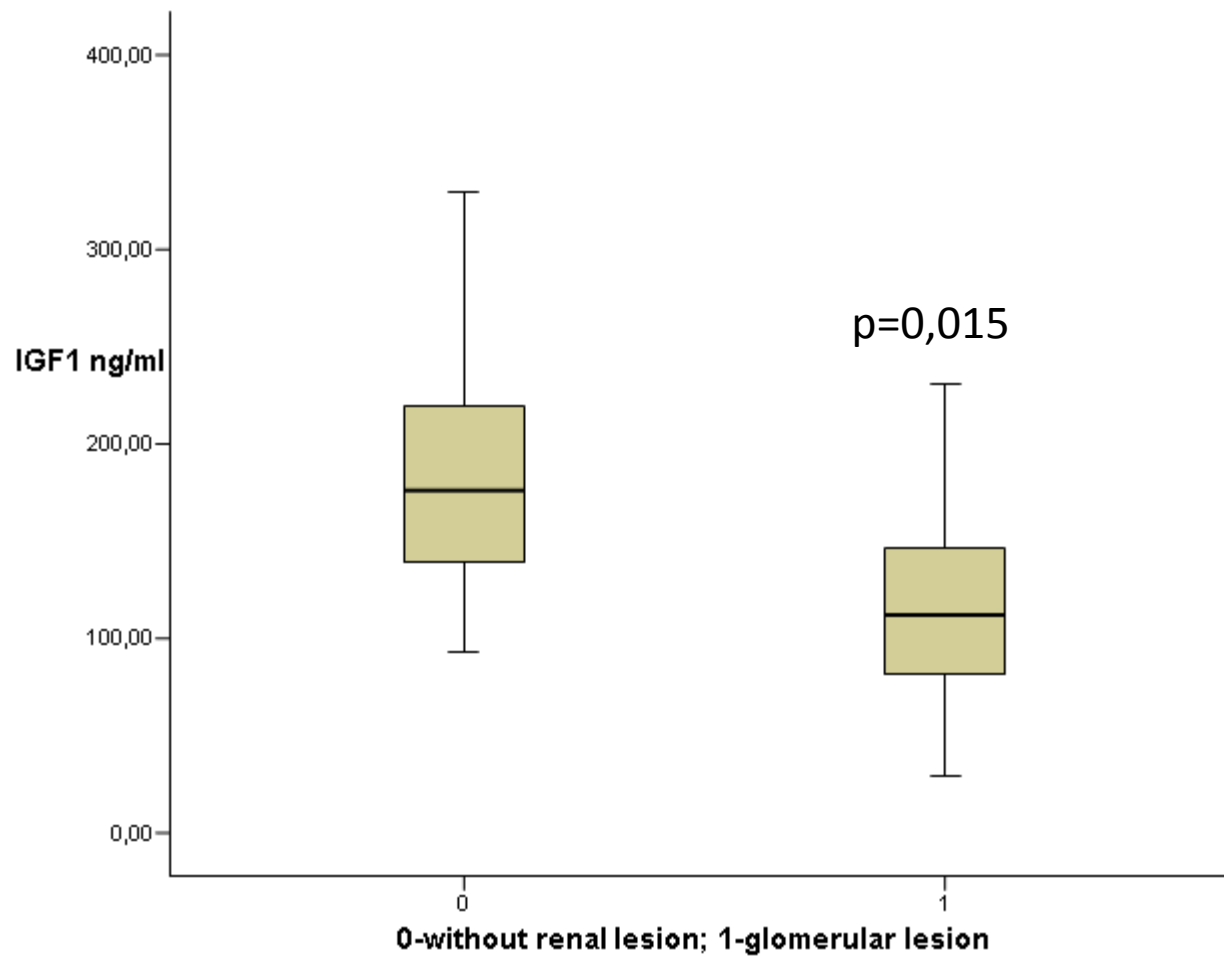

Supplement: S1 Fig — (PDF) [file pone.0154451.s001.pdf]

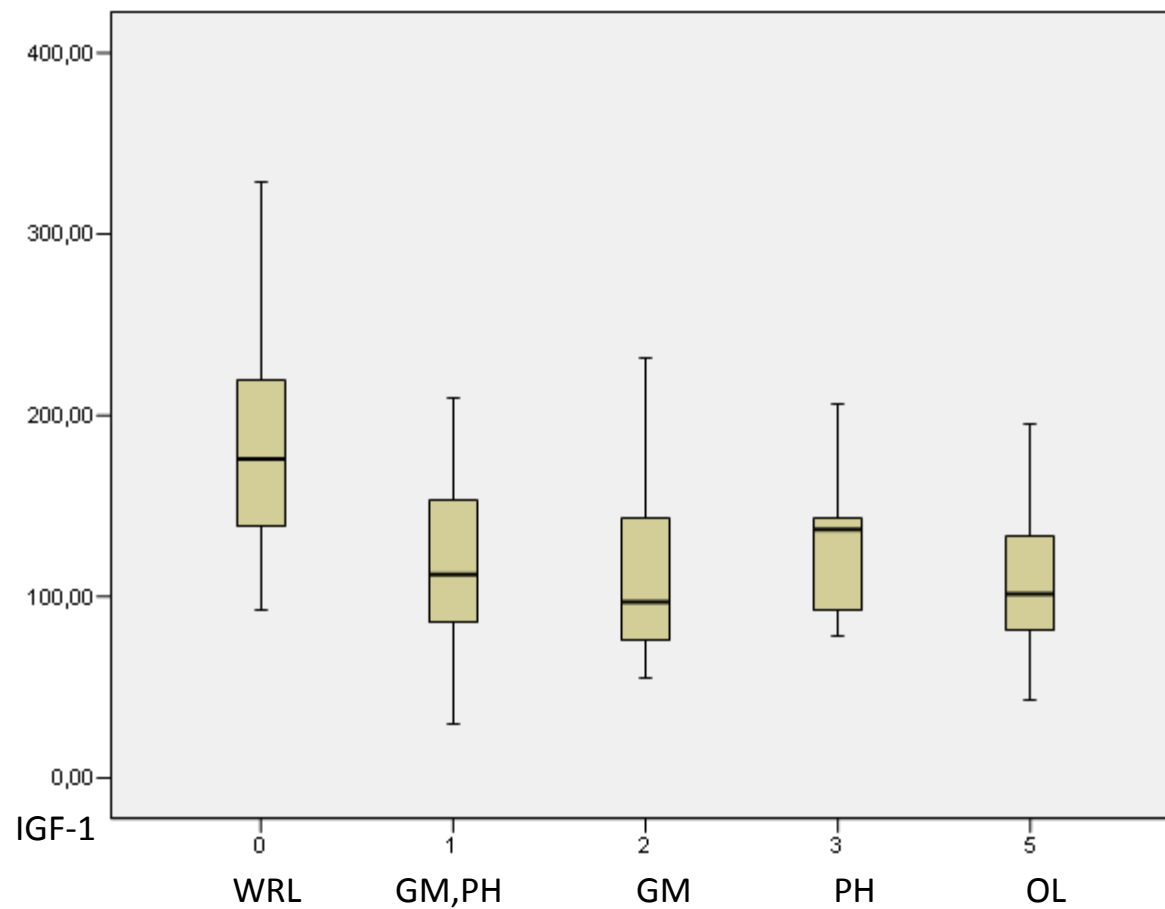

WRL-without renal lesion, GM-glomerulomegaly, PH-podocitary hypertrophy, OL-other lesions

Supplement: S2 Fig — (PDF) [file pone.0154451.s002.pdf]
